# A positive feedback loop involving EGFR/Akt/mTORC1 and IKK/NF-κB regulates head and neck squamous cell carcinoma proliferation

**DOI:** 10.18632/oncotarget.7441

**Published:** 2016-02-17

**Authors:** Zhipeng Li, Zejia Yang, Antonino Passaniti, Rena G. Lapidus, Xuefeng Liu, Kevin J. Cullen, Han C. Dan

**Affiliations:** ^1^ The Marlene & Stewart Greenebaum Cancer Center, University of Maryland School of Medicine, Baltimore, MD, USA; ^2^ Department of Pathology, University of Maryland School of Medicine, Baltimore, MD, USA; ^3^ Department of Pathology, Georgetown University Medical Center, Washington, DC, USA

**Keywords:** head and neck cancer, IKK, NF-kappaB, mTOR, Akt

## Abstract

The overexpression or mutation of epidermal growth factor receptor (EGFR) has been associated with a number of cancers, including head and neck squamous cell carcinoma (HNSCC). Increasing evidence indicates that both the phosphatidylinositol-3-kinase (PI3K)-Akt-mammalian target of Rapamycin (mTOR) and the nuclear factor-kappa B (NF-κB) are constitutively active and contribute to aggressive HNSCC downstream of EGFR. However, whether these two oncogenic signaling pathways exhibit molecular and functional crosstalk in HNSCC is unclear. Our results now reveal that mTORC1, not mTORC2, contributes to NF-κB activation downstream of EGFR/PI3K/Akt signaling. Mechanistically, mTORC1 enhances the inhibitor of nuclear factor kappa-B kinase (IKK) activity to accelerate NF-κB signaling. Concomitantly, activated NF-κB/IKK up-regulates EGFR expression through positive feedback regulation. Blockage of NF-κB/IKK activity by the novel IKKβ specific inhibitor, CmpdA, leads to significant inhibition of cell proliferation and induction of apoptosis. CmpdA also sensitizes intrinsic cisplatin-resistant HNSCC cells to cisplatin treatment. Our findings reveal a new mechanism by which EGFR/PI3K/Akt/mTOR signaling promotes head and neck cancer progression and underscores the need for developing a therapeutic strategy for targeting IKK/NF-κB either as a single agent or in combination with cisplatin in head and neck cancer.

## INTRODUCTION

Head and neck squamous cell carcinoma (HNSCC) is the sixth most common type of cancer in the world [1–3]. Despite the advances in clinical therapy over the last 20 years, it still remains one of the most significant causes of cancer-related death [1–3]. The molecular mechanisms involved in HNSCC initiation, development, progression and therapeutic resistance are associated with the mutation, overexpression or amplification of oncogenic genes and with mutation or loss of function of tumor suppressor genes [2–4]. Mounting evidence indicates that the epidermal growth factor receptor (EGFR) plays critical roles in the pathogenesis and clinical course of HNSCC [2, 5]. In fact, overexpression of EGFR is found in approximately 90% HNSCC [5–9]. Overexpression of EGFR has been associated with poor prognosis, increased tumor growth, metastasis and resistance to chemotherapy and radiation therapy in HNSCC [2, 5, 6]. The overexpressed or activated EGFR in turn activates several critical signaling pathways, with the most frequently altered signaling pathways in HNSCC being the Phosphatidylinositide 3-kinases (PI3Ks)-Akt-Mammalian target of Rapamycin (mTOR) and NF-κB/IKK signaling pathways [2, 5, 6, 10–12].

PI3Ks are a family of enzymes that consist of three different classes: Class I, Class II and Class III. The class IA PI3K is composed of a heterodimer of a p110 catalytic subunit and a p85 regulatory subunit [12–14]. Its most important downstream target is the serine/threonine kinase Akt. Upon activation, PI3K phosphorylates PtdIns(3,4)P2 to produce PtdIns(3,4,5)P3, which leads to translocation of AKT to the plasma membrane, where it is phosphorylated by the phosphoinositide-dependent kinase-1 (PDK1) at threonine 308. Once activated, Akt phosphorylates many downstream substrates to regulate multiple cellular processes including apoptosis, metabolism, cell proliferation and cell growth [12–16]. A major downstream target of Akt is mTOR complex 1 (mTORC1), which is comprised of mTOR, Raptor and GβL [17–20]. mTORC1, phosphorylates its downstream targets 4E-BP1 and S6K and promotes RNA translation [19, 20]. Akt activates mTORC1 through phosphorylation and inhibition of tumor-suppressor protein TSC2 (tuberin) thus releasing TSC2 inhibition of Rheb, which subsequently leads to mTORC1 activation [21–24]. Interestingly, mTOR also associates with Rictor, GβL and mSin1 and forms another mTOR complex, mTORC2, which phosphorylates Akt on serine 473 to fully activate Akt [25–27]. Both mTORC1 and mTORC2 play critical roles in many cancers, but the exact mechanisms by which they regulate different molecular and cellular processes in HNSCC are not well documented. Therefore, it is especially important to identify new critical downstream targets of mTOR in HNSCC.

The transcription factor NF-κB family contains five members: p65 (RelA), RelB, c-Rel, p50/p105 (nuclear factor [NF]-κB1), and p52/p100 (NF-κB2) [28, 29]. In the classical NF-κB signaling pathway, p65 and p50 form a heterodimer that is sequestered in an inactive state by IκBα in cytoplasm. NF-κB is activated by its upstream kinase, which is comprised of two catalytic subunits, IKKα and IKKβ, as well as a regulatory subunit, IKKγ/NEMO [28–32]. Upon stimulation, IKKs phosphorylate IκBα, which leads to their degradation and, eventually, releases IκBα inhibition of p65, causing NF-κB translocation to the nucleus to induce target gene expression. In addition, IKKs phosphorylate RelA/p65 at serine 536, which is associated with transcriptional activity [28–32]. Activated NF-κB is broadly involved in oncogenesis through its ability to promote cell proliferation and to suppress apoptosis. In cancers, the NF-κB signaling pathway also plays a critical role in regulating metastasis, angiogenesis, and chemotherapy resistance [28–32]. Although it is accepted that the NF-κB plays many important roles in cancers, its connection with the other critical oncogenic pathways downstream of EGFR activation in HNSCC is still unclear.

It was reported that there is a significant molecular link between mTORC1 and IKK/NF-κB pathways prostate cancers with PTEN loss and constitutive Akt [33, 34]. In the present study, we investigated the crosstalk between mTORC1 and IKK/NF-κB signaling pathways in HNSCC, which exhibit overexpression of EGFR as well as higher basal levels of activity of both Akt and mTOR [2, 4, 10, 12]. Our data indicate that not only is IKK/NF-κB a critical downstream effector of EGFR/Akt/mTORC1, but it is also an upstream regulator of this pathway.

## RESULTS

### Depletion of mTORC1, rather than mTORC2, impairs NF-κB activity in HNSCC

**Figure 1:**
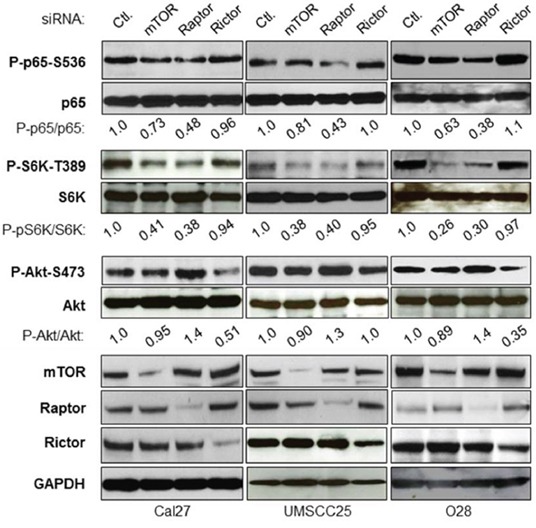
mTORC1, but not mTORC2 involves NF-κB activation in head and neck cancer cells Cells were transfected with siRNA control, or siRNA against mTOR, Raptor or Rictor as indicated. The cells were lysed 72 hours after transfection and the levels of mTOR, Raptor, Rictor, IKKα, IKKβ, S6K and GAPDH, and of endogenous phosphorylation of S6K, p65, and Akt were determined by probing with the indicated antibodies. The results are representative of three experimental repetitions.

The two critical oncogenic pathways, Akt/mTOR and IKK/NF-κB, are constitutively active in both human head and neck cancer specimens as well as cell lines [2, 4, 10, 12]. It would be interesting to define their potential molecular link. We first examined whether or not the mTOR complexes, mTORC1 and mTORC2, are involved in IKK/NF-κB activation in HNSCC. The siRNAs against mTOR, Raptor and Rictor were employed to reduce the expression of both mTORC1 (mTOR and Raptor) and mTORC2 (mTOR and Rictor) in Cal27 cells, an established cell line derived from the poorly differentiated squamous cell carcinoma of the tongue [35]. As shown in Figure 1 (left panel), the expression of mTOR, Raptor and Rictor is markedly reduced upon their siRNA transfection. To test the effects of knockdown of mTOR, Raptor and Rictor on the activity of NF-κB (p65), phosphorylation of NF-κB serine 536, a critical marker for NF-κB activity [36–39], was determined by the specific antibody. The results show that knockdown of mTOR and Raptor, but not Rictor, dramatically impaired p65 phosphorylation at serine 536. Consistent with the function of mTORC1, siRNA to Raptor reduces mTORC1 activity, as shown by the reduction in phosphorylation of S6K, a downstream target of mTORC1. In addition, siRNA to Raptor (mTORC1) leads to upregulation of Akt phosphorylation through the release of mTORC1 feedback inhibition of Akt [40–42]. Furthermore, reduction in the expression of Rictor has no effect on phosphorylation of S6K and NF-κB, although Akt phosphorylation does decrease. Next, mTOR, Raptor and Rictor were knocked down in two other head and neck cancer cell lines, UMSCC25 (Figure 1, middle panel) and O28 (Figure 1, right panel), and its effects on NF-κB, mTOR, and Akt activity were examined. The results showed that knockdown of mTOR and Raptor reduces NF-κB activity, whereas knockdown of Rictor does not have any effect on NF-κB activity. Therefore, these results demonstrate that mTORC1, rather than mTORC2, regulates NF-κB activity downstream of Akt in HNSCC.

### Rapamycin inhibits mTORC1 and NF-κB activity in HNSCC

**Figure 2:**
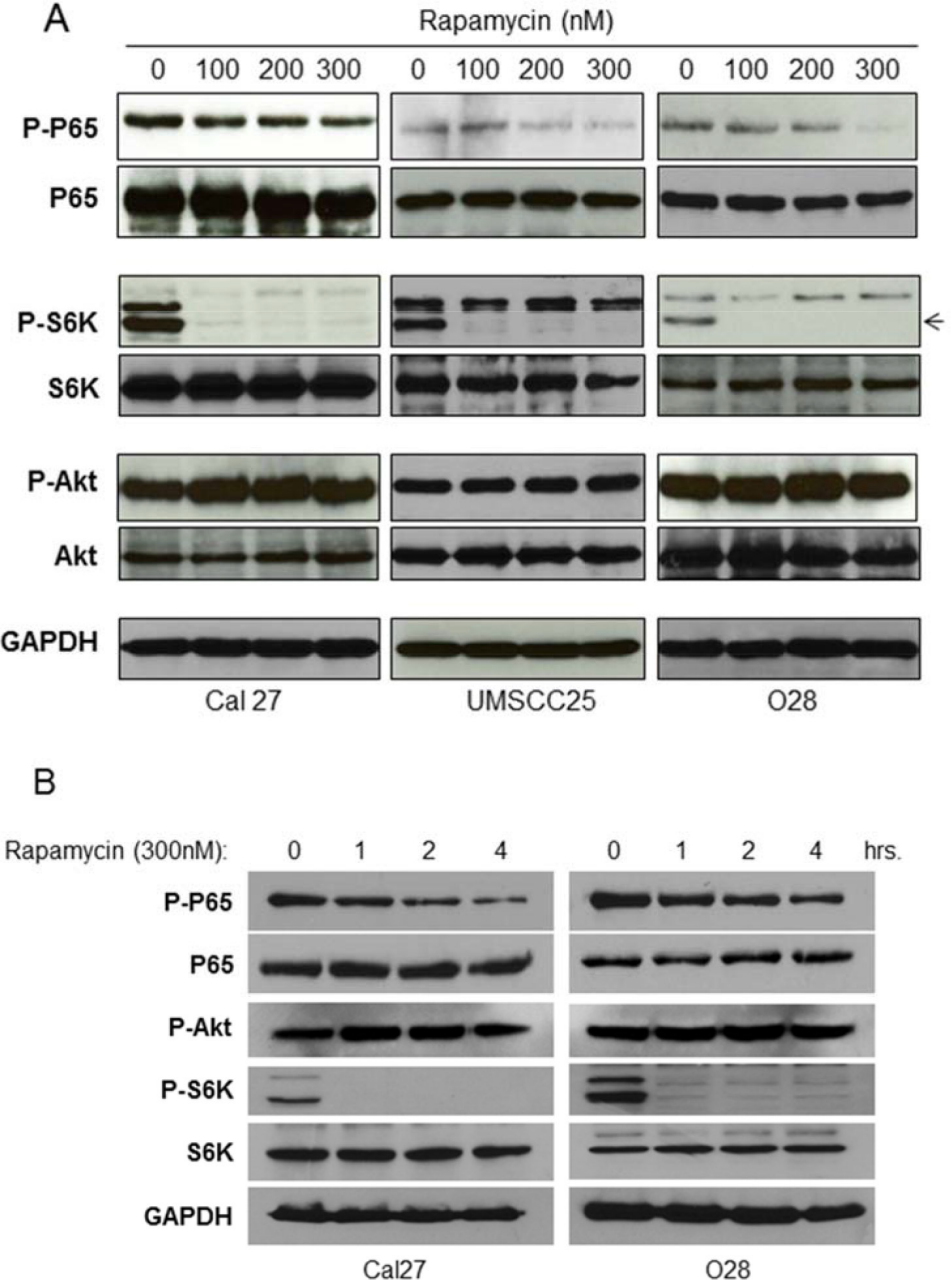
Rapamycin inhibits NF-κB activation HNSCC **A.** Cells were treated with increasing doses of Rapamycin for 1 hour and then cells were lysed. The levels of p65, Akt, S6K and of endogenous phosphorylation of S6K, p65, and Akt were determined by probing with the indicated antibodies. The experiments were repeated for three times. **B.** Cells were treated with 300 nM Rapamycin for 1-4 hours and then cells were lysed for western bot.

If mTORC1 is involved in NF-κB activation, one would expect that treatment with mTORC1 inhibitors, such as Rapamycin, would lead to inhibition of NF-κB activity in these cells. To test this possibility, Cal27, UMSCC25 and O28 cells were treated with Rapamycin at different doses for one hour and the activity of mTOR, Akt and NF-κB were determined by western blot. Rapamycin treatment leads to complete inhibition of phosphorylation of S6K and a slight induction of phosphorylation of Akt, suggesting that Rapamycin inhibits mTOR and subsequently induces Akt through feedback regulation, as described previously [40–42]. Most importantly, Rapamycin greatly limits NF-κB activity (Figure 2A). Furthermore, Rapamycin inhibits NF-κB activity in a time-dependent manner (Figure 2B). These data indicated that Rapamycin inhibits activity of mTORC1 and NF-κB downstream of Akt, which are consistent with the result that mTORC1 induces NF-κB activity downstream of Akt.

### mTORC1 activates IKK complex

**Figure 3:**
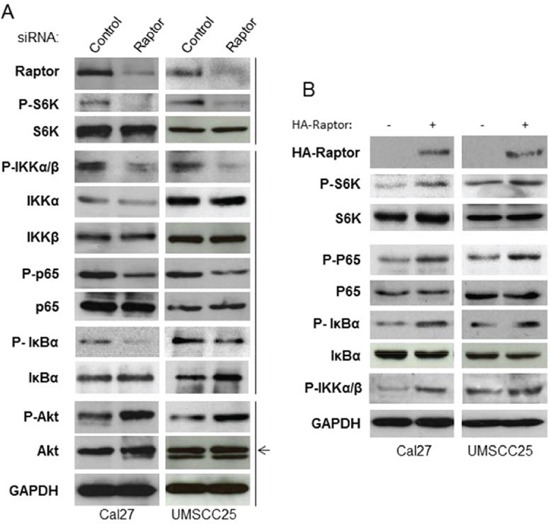
mTORC1 affects IKK activity downstream of Akt and upstream of NF-κB in HNSCC **A.** Cells were transfected with siRNA control, or siRNA against Raptor for 72 hours and then were lysed. The levels of Rpator, p65, IκBα, IKKα, IKKβ, S6K, Akt and GAPDH, and of endogenous phosphorylation of S6K, p65, IκBα and Akt were determined. The results are representative of three experimental repetitions. **B.** HA-tagged Raptor was transfected in the cells and cells lysed for western blot with the indicated antibodies.

A connection between IKK complex and mTORC1 in PTEN null/Akt active prostate cancer has been reported [33, 34]. While HNSCC cells ubiquitously overexpress high levels of phosphorylated Akt, mTOR and NF-κB, it is possible that mTORC1 also enhances IKK activity to promote NF-κB downstream of Akt in HNSCC cells. Thus, we determined whether or not mTORC1 is involved in IKK activation. The siRNA against Raptor was employed to knock down Raptor expression in the Cal27 and SCC25 cells, and their effects on phosphorylation of S6K, Akt and IKKα/β serine 180/181, the marker of IKKα/β activity, were examined. As expected, and consistent with the data shown in Figure 1, knockdown of Raptor decreases phosphorylation of S6K, IκBα and p65, while also increasing phosphorylation of Akt. Most importantly, knockdown of Raptor also leads to dramatically decreased phosphorylation of IKKα/β at serine 177/181 while having no effects on their expression (Figure 3A). To confirm the results of the siRNA knockdown experiments, HA-tagged Raptor was expressed in Cal27 and UMSCC25 cells before the phosphorylation of S6K, IκBα and p65 were determined. Overexpression of Raptor induces the phosphorylation of S6K, IκBα, p65 and IKK (Figure 3B). These results demonstrated that mTORC1 activates NF-κB through activation of IKK complex.

### mTORC1 is required for Akt activation of NF-κB in HNSCC

**Figure 4:**
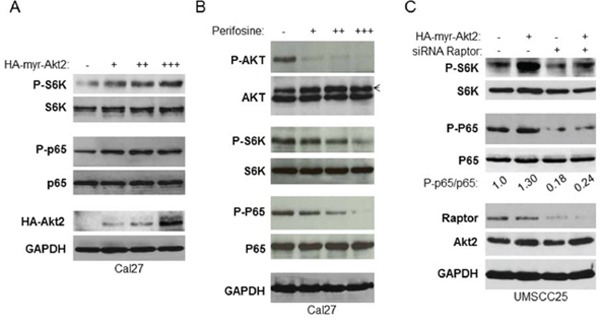
mTORC1 is required for Akt induction of NF-κB in HNSCC **A.** Overexpression of Akt increases activity of mTORC1 and NF-κB. Different doses of active HA tagged Akt2 were transfected in Cal27 cells and phosphorylation p65 and S6K and total p65 and S6K, as well as HA-Akt2 expression were tested by western blot. **B.** Inhibition of Akt, mTORC1 and NF-κB by Akt inhibitor, perifosine. Cells were treated with different doses of perifosine for 24 hours and the levels of p65, Akt, S6K and of endogenous phosphorylation of S6K, p65, and Akt were determined by probing with the indicated antibodies. **C.** Depletion of Raptor blocks Akt induction of NF-κB. Cells were transfected with non-target siRNA or siRNA against Raptor as indicated for 48 hours and active Akt2 were transfected for another 24 hours. Cells were lysed and the levels of p65, Akt, S6K and of endogenous phosphorylation of S6K, p65, Akt2, and Raptor were determined. The results are representative of three experimental repetitions.

Next, we determined whether or not mTORC1 is involved in Akt regulation of NF-κB in HNSCC. HA-tagged active Akt2 (HA-tagged active myr-Akt2) was overexpressed at different doses in Cal27 cells to test their effects on activation of mTOR and NF-κB. The results showed that expression of exogenous HA-Akt2 increases in a dose-dependent manner and is accompanied by increased phosphorylation of both S6K and NF-κB without affecting the total levels of S6K and NF-κB (Figure 4A). Likewise, over-expression of Akt1 up-regulates mTOR and NF-κB (data not shown). Furthermore, we treated Cal27 cells with different concentrations of Akt inhibitor, perifosine (44), for two hours and determined its effects on the basal activity of mTORC1 and NF-κB. The results showed that perifosine completely blocks Akt phosphorylation on serine 473 and dramatically decreases phosphorylation of S6K and NF-κB (Figure 4B). Taken together, our data suggest that Akt activates mTORC1 and NF-κB. Next, we investigated whether mTORC1 is involved in Akt-dependent regulation of NF-κB. UMSCC25 cells were transfected with non-target siRNA control or siRNA against Raptor for 48 hours followed by transfection of vector control or active Akt2 for another 24 hours. The results showed that overexpression of active Akt induced phosphorylation of both S6K and NF-κB in the cells with normal Raptor expression (Figure 4C, compared lanes 1 to lane 2), however, in the Raptor knockdown cells, overexpression of active Akt2 had no effect on phosphorylation of both S6K and NF-κB (Figure 4C, lanes 3 and 4). These data demonstrate that Akt activation of NF-κB occurs through mTORC1 in HNSCC.

### EGFR activation of NF-κB is mTORC1 dependent

**Figure 5:**
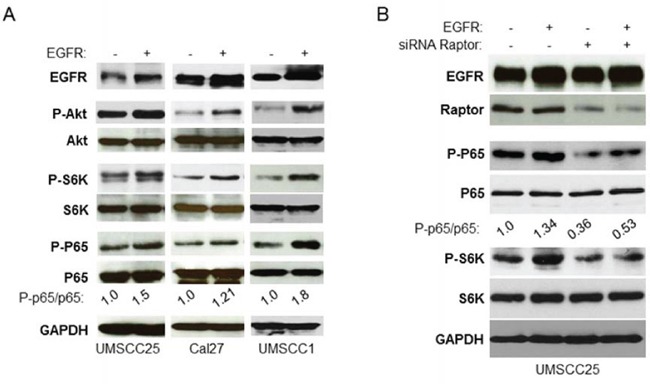
EGFR induction of NF-κB is mTORC1 dependent in HNSCC **A.** Overexpression of EGFR increases activity of Akt, mTORC1, and NF-κB. EGFR was transfected in UMSCC25, Cal27 and UMSCC1 cells and the phosphorylation of Akt, S6K and p65, and total Akt, S6K and p65, as well as EGFR expression were tested by western blot. **B.** Depletion of Raptor blocks EGFR induction of NF-κB. Cells were transfected with non-target siRNA or siRNA against Raptor for 48 hours and then EGFR were transfected for another 24 hours. Cells were lysed and the levels of p65, Akt, S6K and of endogenous phosphorylation of S6K, p65, Akt2 and Raptor, as well as total EGFR were determined by probing with the indicated antibodies. All experiments were repeated for three times.

Both PI3K/Akt/mTOR and IKK/NF-κB signaling pathways are activated by EGFR [2, 4, 10, 12]. Hence, we defined whether EGFR activates NF-κB through mTORC1. Wild type EGFR was overexpressed in UMSCC25 and Cal27 cells and its effects on phosphorylation of Akt, S6K and NF-κB were examined by western blot. The results showed that overexpression of EGFR increases phosphorylation of Akt, S6K and NF-κB (Figure 5A). It should be noted that EGRF transfection-induced up-regulation of Akt, mTOR and NF-κB was not strong potentially because of the high level of basal EGFR. We tested EGFR expression in several more HNSCC cell lines and found that UMSCC1 cell line has lower level of EGFR (data not shown). EGFR overexpression caused dramatic elevation of Akt, mTOR and NF-κB in UMSCC1 cells (Figure 5A). Next, we tested whether EGFR activation of NF-κB depends on mTORC1. UMSCC25 cells were transfected with non-target siRNA or siRNA against Raptor for 48 hours followed by transfection of the vector control or EGFR and activity of mTOR and NF-κB was determined. The result showed that overexpression of EGFR increases the levels of phosphorylation of S6K and NF-κB in the non-target siRNA transfected cells but not in the siRNA Raptor transfected cells (Figure 5B). Therefore, these results indicate that EGFR upregulation of NF-κB depends on mTORC1.

### Akt up-regulates EGFR expression through mTORC1 and NF-κB

**Figure 6:**
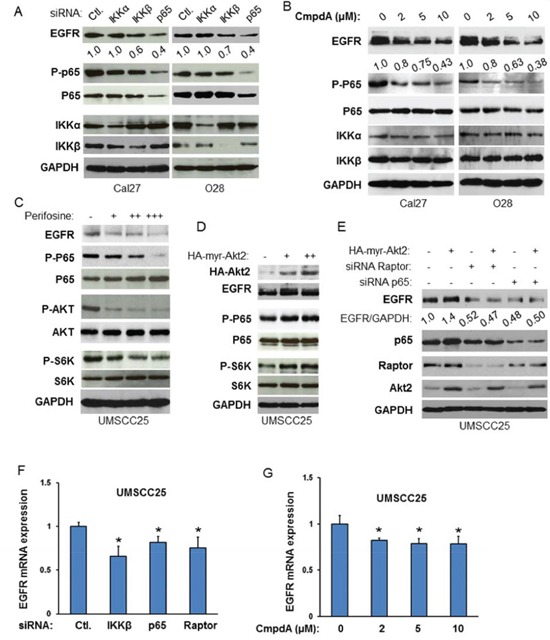
Akt-mTORC1-IKK-NF-κB signaling cascade controls EGFR expression in HNSCC **A.** Cells were transfected with siRNA control, or siRNA against IKKα, IKKβ and p65 as indicated. The cells were lysed 72 hours after transfection and the levels of EGFR, IKKα, IKKβ and p65 and of phosphorylation of p65 were determined by probing with the indicated antibodies. **B.** Cells were treated with different doses of CmpdA for 24 hours. The levels of EGFR, p65, IKKα, and IKKβ, as well as phosphorylation of p65 were determined by western blot. **C.** Cells were treated with different doses of Akt inhibitor, perifosine for 24 hours. The levels of EGFR, p65, Akt and S6K, as well as phosphorylation of p65, Akt and S6K were determined by western blot. **D.** Different doses of active HA tagged Akt2 were transfected in SCC25 cells and the phosphorylation p65 and S6K and total p65 and S6K, as well as EGFR and HA-Akt2 expression were tested by western blot. **E.** Depletion of Raptor or NF-κB blocks Akt induction of EGFR. SCC25 cells were transfected with non-target siRNA or siRNA against Raptor or NF-κB as indicated for 48 hours and then active Akt2 were transfected for another 24 hours. Cells were lysed and the levels of EGFR, Raptor, p65, and Akt2 were determined by probing with the indicated antibodies. All results are representative of three experimental repetitions. **F.** Cells were transfected with control siRNA, or siRNA to IKKβ, p65 or Raptor, as indicated. RNA was extracted 48 h after transfection and RT–PCR (see the Materials and Methods) was performed to assess changes in mRNA levels of EGFR. **G.** Cells were treated with different doses of CmpdA for 48 hours and RNA was extracted for RT–PCR to assess changes in mRNA levels of EGFR.

Our data demonstrated that EGFR/Akt/mTORC1 induces IKK/NF-κB signaling pathway. Interestingly, a recent study by Nottingham and colleagues showed that IKKα and IKKβ synergistically promote the expression and activity of both EGFR and AP1 transcription factors through NF-κB-mediated transcription in head and neck cancer [44]. These observations prompted us to examine whether EGFR/Akt/mTORC1 and IKK/NF-κB pathways are functionally related through interaction of IKK and mTORC1 in HNSCC. We first examined whether or not IKK/NF-κB affects the expression of EGFR in Cal27 cells in which we showed that NF-κB activity is regulated by mTORC1. IKKα, IKKβ and NF-κB were knocked down by siRNA and their effects on the expression of EGFR were determined. The results indicate that the protein levels of IKKα, IKKβ and NF-κB are significantly reduced upon siRNA transfection while knockdown of IKKα or IKKβ decreases phosphorylation of NF-κB p65. Most importantly, decreased EGFR expression is observed after knockdown of IKKβ, and marked decrease of EGRR protein level is observed in the NF-κB p65 knockdown cells (Figure 6A, left panel). We also knocked down the expression of IKKα, IKKβ or NF-κB in O28 cell, a head neck cell line that is relatively resistant to cisplatin treatment (data not shown), and tested the effects on EGFR expression. In line with the results from Cal 27 cells, knockdown of IKKβ or p65 in O28 cell leads to decrease in EGFR expression with a dramatic decrease of EGRR in NF-κB p65 knockdown cells (Figure 6A, right panel). Interestingly, knocking down IKKα in both Cal27 and O28 cells has no effects on EGFR expression (Figure 6A). These results suggest that IKKβ/NF-κB mediate EGFR expression. Next, we tested whether or not inhibition of IKK/NF-κB affects EGFR expression. CmpdA, a novel IKKβ specific inhibitor that blocks NF-κB p65 phosphorylation in several cancer cell lines [45–48], was employed. Both Cal27 and O28 cells were treated with different doses of CmpdA for 48 hours and their effects on NF-κB and EGFR expression were monitored. CmpdA treatment leads to dose-dependent decrease of phosphorylation of NF-κB and expression of EGFR without affecting expression of NF-κB and IKK (Figure 6B). These data indicate that EGFR is a downstream target of IKK/NF-κB in HNSCC.

Next, we determined whether Akt affects EGFR expression. The UM-SCC25 cell line was treated with perifosine, an Akt inhibitor, at different doses for 24 hours and its effects both on EGFR expression as well as Akt, mTOR and NF-κB activity were examined. The results showed that the Akt inhibitor inhibits phosphorylation of Akt, S6K and p65 in a dose dependent manner while having no effect on the expression of these proteins. Interestingly, perifosine treatment also decreases EGFR expression (Figure 6C). In addition, overexpression of Akt increases EGFR level in a dose dependent manner in UMSCC25 cells (Figure 6D). To determine whether Akt modulates EGFR through mTORC1 and NF-κB, it is critical to test whether overexpression of Akt induced-EGFR expression is blocked by depletion of mTORC1 or NF-κB. UMSCC25 cells were transfected with siRNA against Raptor or p65 for 48 hours and active Akt2 was expressed in these cells. The results showed that the expression of active Akt increases EGFR expression (Figure 6E, compare lane 1 to lane 2) and importantly, that it is restrained by knockdown of Raptor or p65 (Figure 6E, lane 3 to lane 4 and lane 5 to lane 6). The results indicate that EGFR expression is regulated by Akt/mTOR/IKK/NF-κB signaling pathways. Therefore, these results demonstrate that EGFR/Akt/mTORC1 and IKK/NF-κB form a positive regulatory loop through IKK in HNSCC.

To determine whether mTORC1 and IKKβ/NF-κB regulate EGFR expression through transcription, the expressions of IKKβ, p65 and Raptor was reduced by siRNA before RNA was extracted for analysis in UMSCC25 cells. The results showed that knockdown of any of these factors leads to a 20-30% decrease in EGFR expression (Figure 6F). Furthermore, 48 hours of treatment for the IKKβ inhibitor CmpdA in UMSCC25 cells caused a roughly 20% decrease in EGFR expression (Figure 6G). These data suggest that mTORC1 and IKKβ/NF-κB regulation of EGFR expression involves transcription. It should also be noted that IKKβ/NF-κB-mediated EGFR changes at the mRNA level are weaker than those at the protein level, suggesting that mTOR and IKKβ/NF-κB may also regulate EGFR through transcription-independent mechanisms.

### IKKβ inhibitor, CompA suppresses cell proliferation and induces apoptosis in HNSCC

**Figure 7:**
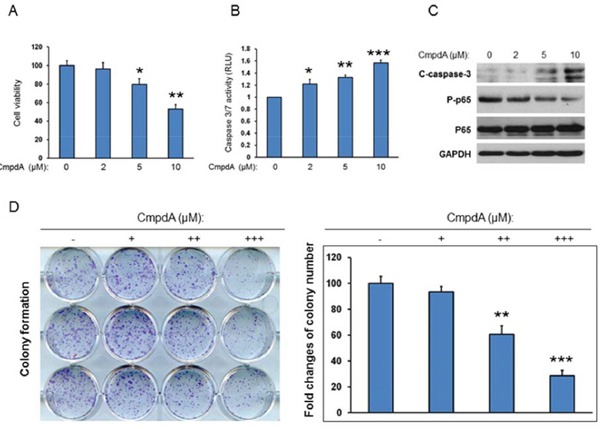
IKK/NF-κB mediates cell proliferation and survival in HNSCC **A.** Cells were treated with different doses of CmpdA for 48 hours and cell viability was measured by MTS assay. The experiments were performed in triplicate and the results are representative of three independent experiments (*, *P* < 0.05, **, *P* < 0.01). **B.** Cells were treated with different doses of CmpdA for 48 hours and caspase activity was measured. The experiments were performed in triplicate, and the results are representative of three independent experiments (**, *P* < 0.01, ***, *P* < 0.001). **C.** Cells were treated with different doses of CmpdA for 48 hours and caspase-3 cleavage was measured by western blot. The results are representative of three independent experiments. **D.** Cells were treated with different doses of CmpdA for 10 days and colony formation was observed and counted. The results are representative of three independent experiments.

Our data demonstrate that IKK/NF-κB is activated downstream of EGFR/Akt/mTORC1 and that IKK/NF-κB positively regulates EGFR levels through feedback regulation. The data also indicate that IKKβ/NF-κB is a key player in the functional interaction of these two critical oncogenic pathways. Therefore, to determine the impact of inhibition of IKK/NF-κB signaling on cell proliferation and survival, we treated HNSCC cells with the IKKβ inhibitor, CmpdA. We have already shown that CmpdA significantly blocks NF-κB activity in Cal27 cells. Cal27 cells were treated with different doses of CmpdA (2-10 μM) for 48 hours and cell proliferation was measured with MTS assay. As shown in Figure 7A, treatment with 2 μM CmpdA causes slight (5%) inhibition of cell proliferation and 5 μM CmpdA leads to higher (20%) inhibition, whereas treatment with 10 μM CmpdA shows significant inhibition (50%). We also examined whether CmpdA induces apoptosis in Cal27 cells. Cells were treated with CmpdA and caspase3/7 activity was measured. The results show that CmpdA induces caspase-3/7 activity in a dose dependent manner (Figure 7B). Consistent with the caspase activity data, western blot analysis showed that CmpdA induces caspase-3 cleavage in a dose dependent manner (Figure 7C). Finally, the inhibitory effects of CmpdA on colony formation were determined. The results showed that CmpdA caused significant reduction of the numbers and sizes of colonies in a dose dependent manner (Figure 7D). Furthermore, we found that CmpdA inhibits cell proliferation and induces apoptosis in two other HNSCC cell lines, SCC25 and FuDa (data not shown). Therefore, IKKβ/NF-κB inhibition induces apoptosis and inhibits cell survival in HNSCC.

### IKKβ inhibitor, CmpdA, improves the efficacy of cisplatin in intrinsic cisplatin resistant HNSCC cells

**Figure 8:**
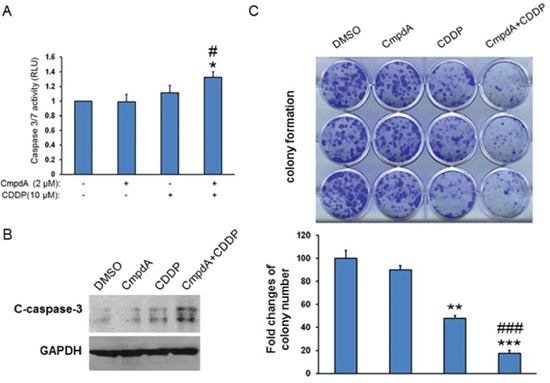
IKKβ inhibitor, CmpdA sensitizes O28 cells to cisplatin-induced apoptosis **A.** Cells were treated with DMSO, CmpdA, cisplatin or CmpdA plus cisplatin for 48 hours and caspase activity was measured. The experiments were performed in triplicate, and the results are representative of three independent experiments (^#^ *P* < 0.05, compared to CDDP treatment; *P < 0.05, compared to DMSO control or CmpdA treatment). **B.** Cells were treated as A for 48 hours and caspase-3 cleavage was determined by western blot. The experiments were repeated three times. **C.** Cells were treated with CompA, Cisplatin, or CompA and Cisplatin as indicated and colony formation was observed 10 days after treatment. Each experiment was repeated three times (^###^ *P* < 0.001, compared to CDDP treatment; **P < 0.01, ***P < 0.001, compared to DMSO control or CmpdA treatment).

Cisplatin is one of the most common antitumor drugs in the treatment of the advanced cancers, including head and neck cancer, but its efficacy is limited due to both intrinsic and acquired resistance, as well as toxicity [49–51]. We tested the sensitivity of a set of head and neck cell lines to cisplatin treatment by MTT assay and noted that the O28 cell line is relatively resistant to cisplatin with an IC50 value at 18 μM. Therefore, we used the O28 cell line to test whether CmpdA sensitizes cisplatin resistant cells to cisplatin treatment. O28 cells were treated with DMSO, CmpdA, cisplatin, or a combination of CmpdA and cisplatin and caspase 3/7 activity was measured. As shown in Figure 8A, a lower dose of compA (2 μM) is not able to induce apoptosis and 10 μM cisplatin leads to slight induction of apoptosis, whereas a combination of CmpdA and cisplatin causes a significant increase in apoptosis (Figure 8A). In a parallel experiment, caspase-3 cleavage was detected by Western blot (Figure 8B). The results show that CmpdA alone did not induce caspase-3 cleavage and cisplatin alone induced minimal induction of caspase-3 cleavage, whereas CmpdA plus cisplatin caused a dramatic induction of caspase-3 (Figure 8B). To further determine the inhibitory effects of these treatments on survival and proliferation, we performed a clonogenic assay with the different treatments. As shown in Figure 8C, the combination of CmpdA and cisplatin demonstrated a significantly reduced number of colonies compared to either agent alone. These results indicate that CmpdA sensitizes intrinsic cisplatin resistant O28 cells to cisplatin treatment.

## DISCUSSION

**Figure 9:**
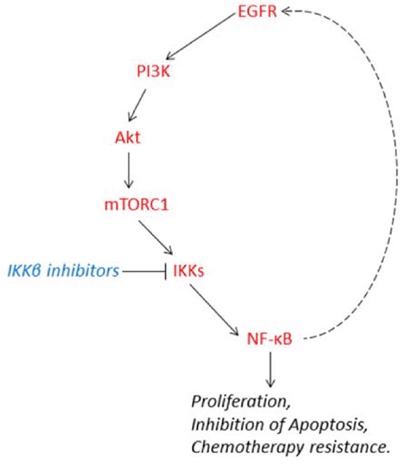
Schematic illustration that IKK/NF-κB forms a positive feedback regulation loop with EGFR/Akt/mTORC1 signaling and mediates cell proliferation, survival and cisplatin resistance in HNSCC

Multiple signaling pathways including PI3K/Akt/mTOR, Jak/STAT3, MEK/ERK and IKK/NF-κB are activated downstream of EGFR in HNSCC [2, 4, 10, 12, 52]. In the current study, we explored the molecular and functional interaction between EGFR/Akt/mTORC1 and IKK/NF-κB pathways in HNSCC. Our data indicate that, first, mTORC1 induces IKK/NF-κB activity in HNSCC. Second, EGFR/Akt regulates IKK/NF-κB signaling through mTORC1. Third, Akt-controlled mTORC1 activation of IKK/NF-κB increases EGFR levels through a positive feedback mechanism. These data suggest that EGFR/Akt/mTOR and IKK/NF-κB pathways form a positive feedback regulation loop in HNSCC and that IKK is the key adaptor in this loop. In addition, IKK/NF-κB plays a critical role in regulation of cell proliferation, survival and intrinsic cisplatin resistance (Figure 9).

It has been reported that Akt activates NF-κB via phosphorylation of IKKα at Threonine 23 [53, 54]. In addition, previous studies demonstrated that mTORC1 contributes to NF-κB activation by interacting with IKK and that this regulation is under the control of Akt in PTEN loss-induced prostate cancer [34]. In the present study, we showed that EGFR-induced Akt activation also regulates mTORC1 and upregulates the IKK/NF-κB pathway. These data suggest that mTORC1 activation of IKK/NF-κB could occur in most cells that have higher basal levels of Akt activity regardless of the tumor type. In addition, our recent study demonstrated that mTORC1 could also negatively affect IKK/NF-κB through feedback regulation in some cell lines that have lower basal activity of Akt (data not shown). It would be interesting to investigate whether mTORC1 affects IKK/NF-κB in other cancer cell lines that have different levels of Akt activity.

mTOC1 is a key downstream target for Akt to regulate tumorigenesis. However, clinical efficacy of mTOR inhibitors including rapamycin has been limited due to feedback up-regulation of PI3K/Akt signaling [41–43]. Our data indicates that rapamycin not only inhibits mTORC1 and IKK/NF-κB signaling but also induces Akt phosphorylation in HNSCC (Figure 2). However, the IKK inhibitor significantly limits both IKK/NF-κB and EGFR/Akt signaling, which suggests that IKK/NF-κB can be an important target in HNCCC. In addition, treatment with IKK inhibitor as a single agent causes significant inhibition of proliferation and induction of apoptosis. It should be stressed that IKK inhibitors in combination with another inhibitor or chemotherapeutic agent may show more benefit in cancer treatment.

The activation of NF-κB in response to chemotherapy is a principal mechanism of inducible tumor chemotherapy resistance [55]. Our data indicate that IKK inhibition by CmpdA dramatically improves the efficacy of cisplatin in overcoming HNSCC cell proliferation and survival. Our observation is in line with the recent studies showing that Curcumin, an antioxidant and anti-inflammatory substance derived from the East Indian plant Curcuma longa, inhibits IKKβ activity and suppresses cell proliferation as a single agent or in combination and cisplatin in HNSCC [56–58]. Therefore, it will be important to test the IKK inhibitor, CmpdA as either a single agent or in combination with other inhibitors or anti-tumor drug in preclinical model of HNSCC *in vivo* and potentially in head and cancer clinical trials.

## MATERIALS AND METHODS

### Cell culture and reagents

Cal 27 cell line was purchased from ATCC. UM-SCC25 cell line and UMSCC1 were the generous gift of T.E. Carey (University of Michigan, Ann Arbor, MI, USA). JHU-O28 cell line was obtained from Dr. Zhongmin Guo and originally from Johns Hopkins University School of Medicine (Baltimore, MD). All cells were maintained in Dulbecco's modified Eagle's medium (DMEM) supplemented with 10% fetal bovine serum (FBS), 2 mM glutamine, and 100 U/mL penicillin and streptomycin (Gibco). The reagents were obtained from the following sources: Protease and phosphatase inhibitor cocktails were from Roche; CHAPS was from Pierce. Akt inhibitor, perifosine is from AdooQ (Catalog No. A10709).

### Cell lysis and western blot analysis

Cells were lysed and immunoblotted as described previously [19, 34]. Briefly, cells grown on 100-mm dishes were rinsed twice with 1x cold PBS and then lysed on ice for 20 min in 1 mL of lysis buffer (40 mM Hepes at pH 7.5, 120 mM NaCl, 1 mM EDTA, 10 mM pyrophosphate, 10 mM glycerophosphate, 50 mM NaF, 0.5 mM orthovanadate, EDTA-free protease inhibitors [Roche]) containing 0.3% CHAPS. After centrifugation at 13,000*g* for 10 min, lysates containing 20–50 μg of protein were resolved by 4-12% SDS-PAGE, and proteins were transferred to Pure Nitrocellulose Membrane (Bio-Rad), blocked in 5% nonfat milk, and blotted with the indicated antibodies. Densitometric analyses of bands were performed using ImageJ software.

### Antibodies

Antibodies were obtained from the following sources: Antibodies against phospho-p65 (CST-3033), p65 (CST-6956), phospho-IKKα/β (CST-2697), IKKα (CST-2682), IKKβ (CST-8943), phospho-Akt (CST-4508), Akt1 (CST-2938), Akt2 (CST-3063), phospho-S6K (CST-9205), mTOR (CST-2972), cleaved caspase 3 (CST-9664), and GAPDH (CST-5174), are purchased from Cell Signaling. Anti-Raptor ((A300-506A) and anti-Rictor (A300-458A) are from Bethyl. Anti-HA (H6908) is from Sigma. Anti-S6K (SC-8418), C-Myc (SC-40), and EGFR (SC-03) are from Santa Cruz Biotechnology. HRP-labeled anti-mouse and anti-rabbit secondary antibodies were also from Santa Cruz Biotechnology.

### Plasmids and transient transfection

EGFR-expressing plasmid was obtained via Addgene originally from MC Hung. Transfections were performed using Lipofectamine and Plus reagent (Invitrogen) following the manufacturer's instructions. 3–4 h after transfection, the cells were recovered in full serum for 36 h before the assay.

### siRNA and transfection

siRNA SMARTpool IKKα, IKKβ, p65, mTOR, Raptor and Rictor were from Dharmacon. Each of these represents four pooled SMART-selected siRNA duplexes that target the indicated mRNA. Cells were transfected with indicated SMARTpool siRNA or nonspecific control pool using DharmaFECT 1 reagent (Dharmacon) according to the manufacturer's instructions. Briefly, 20 nM final concentration of siRNA was used to transfect cells at 60%–70% confluency. Twenty-four hours after transfection, cells were recovered in full serum. Cells were harvested 48–72 h after siRNA transfection.

### RNA extraction and RT-PCR

RNA was extracted from the cells using Trizol reagent (Life Technologies) and reverse-transcribed following the protocols. Real-time quantitative PCR was carried out with SYBR green mix (Bio-Rad, Hercules, CA) using *CFX96 real*-*time PCR detection system* (*Bio*-*Rad*). EGFR: forward, 5′-AGGCACGAGTAACAAGCTCAC-3′ and reverse, 5′-ATGAGGACATAACCAGCCACC-3′; GAPDH: forward, 5′-ACAACTTTGGTATCGTGGAAGG-3′ and reverse, 5′-GCCATCACGCCACAGTTTC-3′.

### MTS cell proliferation assays

Cells were seeded in 96-well plate in triplicate at 3 × 10^3^ per well and cultured in the presence or absence of cisplatin or the IKKβ inhibitor at the indicated concentrations and time course. At the end of each time point, 3-(4,5-dimethylthiazol-2-yl)-5-(3-carboxymethoxyphenyl)-2-(4-sulfophenyl)-2*H*-tetrazolium (MTS) compound (Promega) was added for 1 hours at 37°C. Colorimetric readouts were read at 490 nm on a Versamax Microplate Reader (Molecular Devices).

### Caspase activity

Cells were plated in triplicate at 2 × 10^3^ per well in white-walled 96-well plates (Becton Dickinson) for 24 hours and then were treated with the IKK inhibitor and/or cisplatin for additional 48 hours. Caspase-3/7 activity was measured using the Caspase-Glo 3/7 assay (Promega) according to the manufacturer's instructions. Caspase-Glo 3/7 assay uses a caspase-3/7 tetrapeptide DEVD substrate that produces a luminescent signal on cleavage. Relative light units were measured on an Lmax Microplate Luminometer (Molecular Devices).

### Colony focus assay

Cells were plated the day before treatment at 2000 cells per well in a six-well plate. The next day, cells were pre-treated with DMSO or CmpdA for 2 hours and then treated with cisplatin for another 2 hours. After the two hour incubation with cisplatin, medium was replaced with DMSO or CmpdA containing media and cells were allowed to grow. Cells were allowed to form colonies for 10 days. The plates were then gently washed with phosphate-buffered saline and colonies stained with crystal violet. Each experiment was repeated three times.

### Statistics

Data from the *in vitro* experiments are expressed as mean ± SD from a minimum of 3 independent experiments. Comparison between groups were carried out by 2-way ANOVA or Student *t* test, and a *P* value of less than 0.05 was considered significant.
